# A Randomized, Double-Blind, Phase 3 Safety and Efficacy Study of Ridinilazole Versus Vancomycin for Treatment of *Clostridioides difficile* Infection: Clinical Outcomes With Microbiome and Metabolome Correlates of Response

**DOI:** 10.1093/cid/ciad792

**Published:** 2024-02-02

**Authors:** Pablo C Okhuysen, Mayur S Ramesh, Thomas Louie, Nino Kiknadze, Julian Torre-Cisneros, Claudia Murta de Oliveira, Christophe Van Steenkiste, Alena Stychneuskaya, Kevin W Garey, Julia Garcia-Diaz, Jianling Li, Esther Duperchy, Betty Y Chang, Juthamas Sukbuntherng, Jose G Montoya, Lori Styles, Fong Clow, Danelle James, Erik R Dubberke, Mark Wilcox

**Affiliations:** Department of Infectious Diseases, Infection Control, and Employee Heatlh, The University of Texas MD Anderson Cancer Center, Houston, Texas, USA; Henry Ford Health, Detroit, Michigan, USA; Foothills Medical Center and University of Calgary, Calgary, Canada; Aversi Clinic, Tbilisi, Georgia; Reina Sofia University Hospital-IMIBIC, University of Córdoba, CIBERINFEC, Cordoba, Spain; Santa Casa de Belo Horizonte, Belo Horizonte, Brazil; Algemeen Ziekenhuis Maria Middelares, Ghent, Belgium; University Antwerp,Antwerp, Belgium; Vitebsk Regional Clinical Hospital of Infectious Diseases, Vitebsk, Belarus; University of Houston College of Pharmacy, Houston, Texas, USA; Ochsner Health, New Orleans, Louisiana, USA; Summit Therapeutics, Menlo Park, California, USA; Summit Therapeutics, Menlo Park, California, USA; Summit Therapeutics, Menlo Park, California, USA; Summit Therapeutics, Menlo Park, California, USA; Summit Therapeutics, Menlo Park, California, USA; Dr. Jack S. Remington Laboratory for Specialty Diagnostics, Palo Alto Medical Foundation, Palo Alto, California, USA; Summit Therapeutics, Menlo Park, California, USA; Summit Therapeutics, Menlo Park, California, USA; Summit Therapeutics, Menlo Park, California, USA; Washington University School of Medicine, St.Louis, Missouri, USA; Leeds Teaching Hospitals and University of Leeds, School of Medicine, Leeds, United Kingdom

**Keywords:** ridinilazole, vancomycin, *Clostridioides difficile*, microbiome, bile acids

## Abstract

**Background:**

Exposure to antibiotics predisposes to dysbiosis and *Clostridioides difficile* infection (CDI) that can be severe, recurrent (rCDI), and life-threatening. Nonselective drugs that treat CDI and perpetuate dysbiosis are associated with rCDI, in part due to loss of microbiome-derived secondary bile acid (SBA) production. Ridinilazole is a highly selective drug designed to treat CDI and prevent rCDI.

**Methods:**

In this phase 3 superiority trial, adults with CDI, confirmed with a stool toxin test, were randomized to receive 10 days of ridinilazole (200 mg twice daily) or vancomycin (125 mg 4 times daily). The primary endpoint was sustained clinical response (SCR), defined as clinical response and no rCDI through 30 days after end of treatment. Secondary endpoints included rCDI and change in relative abundance of SBAs.

**Results:**

Ridinilazole and vancomycin achieved an SCR rate of 73% versus 70.7%, respectively, a treatment difference of 2.2% (95% CI: −4.2%, 8.6%). Ridinilazole resulted in a 53% reduction in recurrence compared with vancomycin (8.1% vs 17.3%; 95% CI: −14.1%, −4.5%; *P* = .0002). Subgroup analyses revealed consistent ridinilazole benefit for reduction in rCDI across subgroups. Ridinilazole preserved microbiota diversity, increased SBAs, and did not increase the resistome. Conversely, vancomycin worsened CDI-associated dysbiosis, decreased SBAs, increased Proteobacteria abundance (∼3.5-fold), and increased the resistome.

**Conclusions:**

Although ridinilazole did not meet superiority in SCR, ridinilazole greatly reduced rCDI and preserved microbiome diversity and SBAs compared with vancomycin. These findings suggest that treatment of CDI with ridinilazole results in an earlier recovery of gut microbiome health.

**Clinical Trials Registration.**Ri-CoDIFy 1 and 2: NCT03595553 and NCT03595566.

## Teaser

In this phase 3 superiority study, ridinilazole, bactericidal bis-benzimidazole against *C. difficile*, versus vancomycin reduced recurrent disease by 53%, preserved microbiota diversity, increased secondary bile acids, did not increase resistome, but did not meet superiority in initial clinical response.

## Graphical Abstract

**Graphical Abstract:**
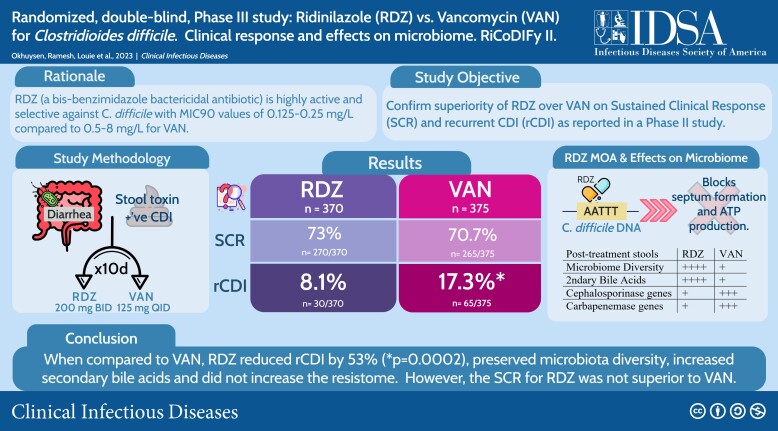
https://tidbitapp.io/tidbits/test-7e5035e4-ad1b-417a-bee9-788b33377fe0

*Clostridioides difficile* infection (CDI) remains the most common healthcare-associated infection reported in the United States and is associated with significant morbidity and emotional and financial hardship [1–4]. Antibiotic-induced perturbations in the colon microbiome and bile acid (BA) composition are crucial events in the pathogenesis of CDI [5–8]. Dysbiosis causes a decrease in secondary BAs (SBAs) that facilitates *C. difficile* spore germination [9–11], favoring enterotoxin-producing vegetative forms that cause diarrhea and/or colitis [12]. Single or multiple episodes of recurrent CDI (rCDI) occur in 25% of patients following a primary CDI [13, 14]. This can be overwhelming to patients and taxes healthcare systems [15]. Clinically available drugs (metronidazole, vancomycin, fidaxomicin) can successfully treat most patients but do not prevent all recurrences [13]. An effective therapy that treats CDI while preventing rCDI is lacking.

Ridinilazole (RDZ) is a bis-benzimidazole bactericidal antibiotic [16, 17] that preferentially binds to AATTT-rich sequences in the *C. difficile* DNA minor groove impacting downstream cell septum formation and, likely, the ability to generate ATP [18]. In vitro, RDZ exhibits a narrow spectrum of activity and is highly active against *C. difficile.* Ridinilazole displays targeted activity against *C difficile* with minimal inhibitory concentration 90 values of 0.125–0.25 mg/L compared to 0.5–8 mg/L for vancomycin (VAN) [17, 19, 20]. Like VAN, RDZ has no activity against aerobic gram-negative bacteria. Unlike VAN, RDZ has no activity against *Enterococcus faecalis*, *Streptococcus*, and other gram-positive anaerobes such as *Clostridium perfringens, Eggerthela*, and *Finegoldia magna* [19, 21]. In clinical trials, analysis of post-treatment fecal microbiota shows that, when compared with RDZ, treatment with VAN results in profound losses of *Bacteroides*, *Clostridium coccoides*, *Clostridium leptum*, and *Prevotella* and expansion of *Enterobacteriaceae*. Ridinilazole selectivity preserves key components of the human gut microbiota in patients with CDI [19, 21], resulting in fewer rCDIs [20].

Based on promising data from a phase 2 study [20], 2 phase 3 studies comparing the efficacy and safety of RDZ with VAN for the treatment of CDI were carried out.

## METHODS

### Trial Design and Oversight

Two phase 3, global, randomized, double-blind, active-controlled clinical trials (Ri-CoDIFy 1 and 2; NCT03595553 and NCT03595566) were conducted at 157 sites, in 26 countries, from 31 January 2019 through 19 November 2021, in accordance with the principles of the Declaration of Helsinki and Good Clinical Practices. To minimize the potential, unknown impact of the continuing coronavirus disease 2019 (COVID-19) pandemic on the trial, the 2 studies were merged, and the statistical analysis conducted on a single dataset that combined both studies. Study protocols and amendments were approved by a central or local institutional review boards. All study patients provided written informed consent prior to enrollment. The first draft of the manuscript was written by employees of Summit Therapeutics and the first author. All authors had access to the data, participated in reviewing and editing of the manuscript, and endorsed the accuracy and integrity of the data.

### Study Population

Eligible patients were 18 years of age or older with CDI, defined by the presence of symptoms and signs including diarrhea (≥3 unformed bowel movements [UBMs] in the 24 hours before randomization) and the presence of *C. difficile* toxin A and B, or B alone, in stools as detected onsite by a US Food and Drug Administration (FDA)– or European Union–approved assay. The stool sample must have been produced less than 72 hours before randomization. Patients were excluded if they had more than 1 episode of CDI within the last 3 months or more than 3 CDI episodes within the last 12 months (complete eligibility criteria included in the Supplementary Methods).

### Randomization and Treatment

Patients were randomized in a 1:1 ratio to receive either RDZ or VAN. Patients were stratified by age (<65 years and ≥65 years) and history of CDI (none or 1 to 3 previous rCDIs in the past 12 months). Patients received the study medication according to a 4-times-per-day regimen for 10 days: 200 mg of RDZ twice a day or 125 mg of VAN 4 times a day, with intervening matching doses of a dummy-placebo in both arms (Supplementary Figure 1).

### Response Definitions

Clinical response was defined as patients passing fewer than 3 UBMs for 2 consecutive days and maintaining through the end of treatment (EOT) without further CDI treatment at EOT + 2 days, or the investigator's assessment that the subject was cured and no longer needed specific CDI antimicrobial treatment after completion of the course of study medication. Recurrent CDI was defined as a new episode of diarrhea (≥3 UBMs) in a 1-day period with a positive *C. difficile* free toxin test or cell cytotoxicity neutralization assay (CCNA) that required CDI treatment in subjects who achieved clinical response. Sustained clinical response (SCR) was defined as clinical response and no rCDI through 30 days post-EOT (day 40 [D40]).

### Outcomes

#### Efficacy Evaluation

The primary endpoint was SCR (D40). Secondary endpoints included clinical response, recurrence, and SCR (days 70 and 100).

#### Gut Bile Acids and Microbiome Analyses

An additional predefined secondary endpoint included change in the relative abundance of microbiome-derived SBAs in stool samples from baseline to EOT. Exploratory endpoints included changes in relative abundance of primary, conjugated primary BAs, and SBAs and in microbiome composition in stool samples at days 40, 70, and 100. Bile acid and microbiome results up to D40 are presented here because the primary endpoint is determined at D40.

Stool collection is described in Supplementary Methods 1. Stool BAs were measured using liquid chromatography with tandem mass spectrometry (Supplementary Methods 2). Microbiome studies were performed using whole-metagenomic deep shotgun sequencing [22] (Supplementary Methods 3).

### Safety

Safety was assessed from the day the informed consent was signed through the end of study visit for all subjects who received at least 1 dose of study treatment. Adverse events were categorized according to the definitions used in the *Medical Dictionary for Regulatory Activities* (MedDRA), version 24.0.

### Statistical Analysis

Efficacy, stool BA, and microbiome composition analyses were based on the modified intent-to-treat (mITT) population. The mITT population was composed of all randomized and treated patients who had 3 or more UBMs in the 24 hours prior to randomization and a diagnosis of confirmed *C. difficile* infection.

The SCR rate was compared between the 2 treatment groups using Cochran-Mantel-Haenszel chi-square test, adjusted for the 2 randomization stratification factors described above. The treatment difference (RDZ vs VAN) and 95% confidence interval (CI) for clinical response rate was calculated based on the stratified Miettinen and Nurminen method [23]. Noninferiority in clinical response would be established if the lower limit of the 2-sided 95% CI for the treatment difference was greater than −10%. Recurrence rate was compared between the 2 treatment groups using a chi-square test. The treatment difference and 95% CI were calculated based on the Miettinen and Nurminen method without stratification factors [23]. Wilcoxon rank-sum test was used to assess change from baseline in BA composition and the different microbiome endpoints within each treatment group, while Wilcoxon rank-sum test was used for comparison between RDZ and VAN groups. The *P* values were corrected for multiple testing using the Benjamini–Hochberg method to control the false discovery rate (FDR) at a level of 10% FDR when comparing relative abundance of microbial taxa and of antibiotic class resistance genes (RGs).

## RESULTS

**Figure 1.**
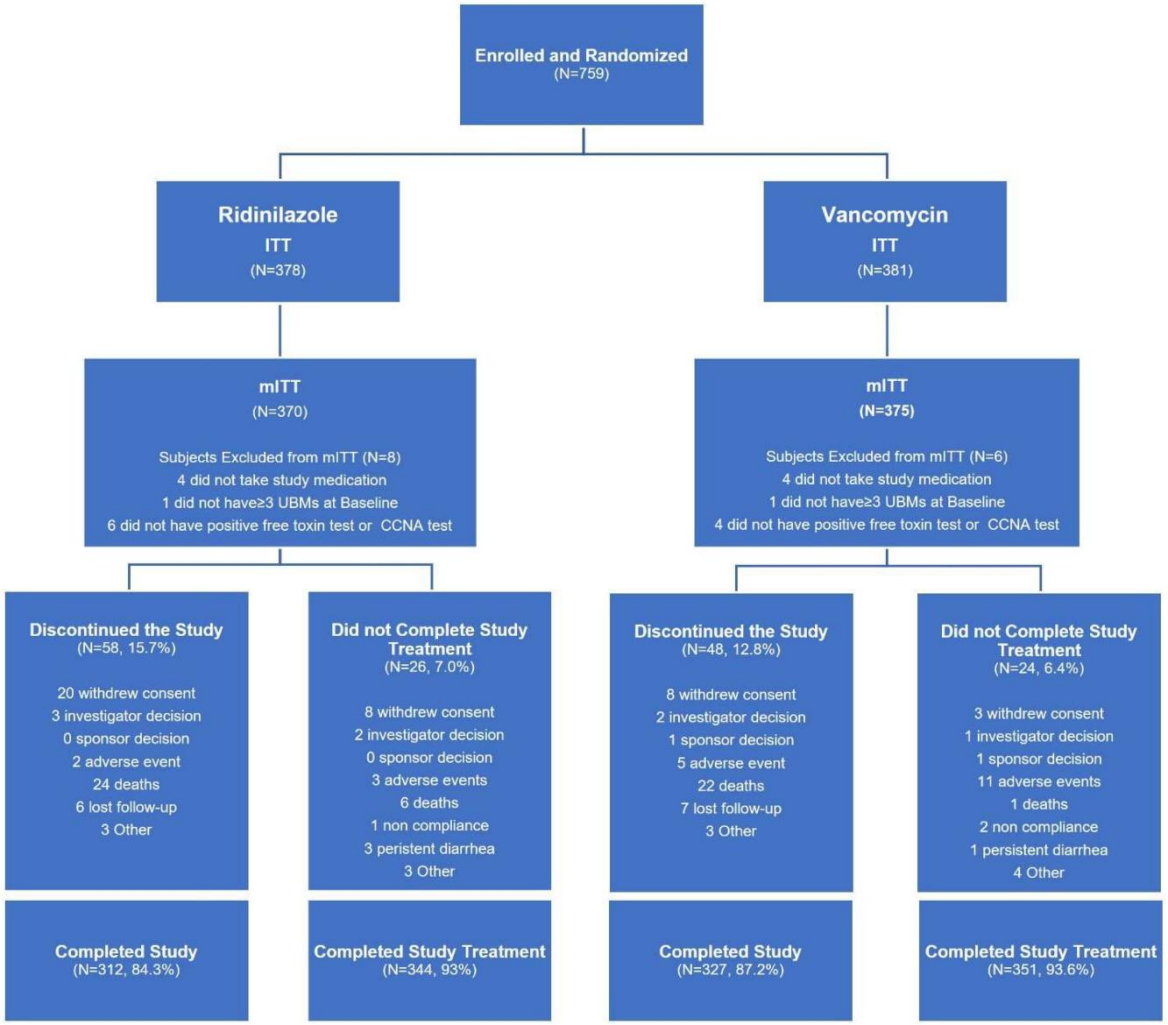
Participant enrollment, randomization, and follow-up. *Subjects could have more than 1 reason for exclusion from the mITT population. The ITT population consists of all randomized subjects and is summarized by randomized treatment assignment; the safety population consists of all subjects who took at least 1 dose of study treatment and is summarized by the actual treatment received by subjects; the mITT population consists of all randomized and treated subjects who had ≥3 UBMs 24 hours prior to randomization and had positive toxin test or CCNA test at baseline and is summarized by randomized treatment assignment. Abbreviations: CCNA, cell cytotoxicity neutralization assay; ITT, intent-to-treat; mITT, modified intent-to-treat; UBM, unformed bowel movement.

Of the 759 subjects who were enrolled and randomized, 745 (98%) were included in the mITT population. Figure 1 depicts enrollment, randomization, and follow-up of participants. In the mITT population, 639 (86%) subjects completed the study. The most common reasons for early discontinuation were death (6% in both treatment groups), withdrawal of consent (5% [20/370] in RDZ and 2% [8/375] in VAN), and loss to follow up (2% in both treatment groups). The median age of subjects in the mITT population was 62 years (range: 18 to 98); 89% were White and 59% were females. Demographic and baseline characteristics were balanced among the 2 treatment groups (Table 1). The median duration of study treatment was 10 days (interquartile range: 9.0–10.0), with a mean relative dose intensity of 94% active doses taken in both treatment groups. The safety population included 751 patients: 374 in the RDZ arm and 377 in the VAN arm. Of note, CCNA testing was performed on 57 stool samples from 54 patients at different time points. The proportion of patients tested by CCNA was too small to generate a separate analysis.

**Table 1.** Demographic and Baseline Characteristics

	RDZ (n = 370), n (%)	VAN (n = 375), n (%)	Total (N = 745), n (%)
Age, median, y	61.0	63.0	62.0
≥65 y	161 (43.5)	162 (43.2)	323 (43.4)
≥75 y	78 (21.1)	83 (22.1)	161 (21.6)
Sex—female	209 (56.5)	227 (60.5)	436 (58.5)
Race			
White	333 (90.0)	331 (88.3)	664 (89.1)
Region			
USA/Canada	93 (25.1)	104 (27.7)	197 (26.4)
Europe	229 (61.9)	220 (58.7)	449 (60.3)
History of prior episodes of CDI in last 12 months			
None	308 (83.2)	309 (82.4)	617 (82.8)
1 Previous episode	59 (15.9)	63 (16.8)	122 (16.4)
2 Previous episodes	2 (0.5)	2 (0.5)	4 (0.5)
3 Previous episodes	1 (0.3)	1 (0.3)	2 (0.3)
Number of UBMs at baseline			
Median (minimum, maximum)	6 (3, 30)	6 (3, 30)	6 (3, 30)
IDSA severity			
Nonsevere	256 (69.2)	265 (70.7)	521 (69.9)
Severe	95 (25.7)	88 (23.5)	183 (24.6)
Disease severity (UBM and WBC criteria)			
Mild	136 (36.8)	131 (34.9)	267 (35.8)
Moderate	105 (28.4)	109 (29.1)	214 (28.7)
Severe	113 (30.5)	114 (30.4)	227 (30.5)
Hospitalization status			
Inpatient	204 (55.1)	196 (52.3)	400 (53.7)
Outpatient	166 (44.9)	179 (47.7)	345 (46.3)
Presence of hypervirulent strain^a^	70 (18.9)	81 (21.6)	151 (20.3)
Presence of ribotype 027 strain	34 (9.2)	47 (12.5)	81 (10.9)
No treatment for current CDI episode	329 (88.9)	328 (87.5)	657 (88.2)
Non-CDI antibiotic usage at baseline			
Yes	115 (31.1)	109 (29.1)	24 (30.1)
No	255 (68.9)	266 (70.9)	521 (69.9)

Abbreviations: CDI, *Clostridioides difficile* infection; IDSA, Infectious Diseases Society of America; RDZ, ridinilazole; UBM, unformed bowel movement; VAN, vancomycin; WBC, white blood cell count.

^a^Hypervirulent ribotypes included ribotypes 027, 078, 126, 176, 198, 244, and 023.

### Clinical Outcomes

**Figure 2.**
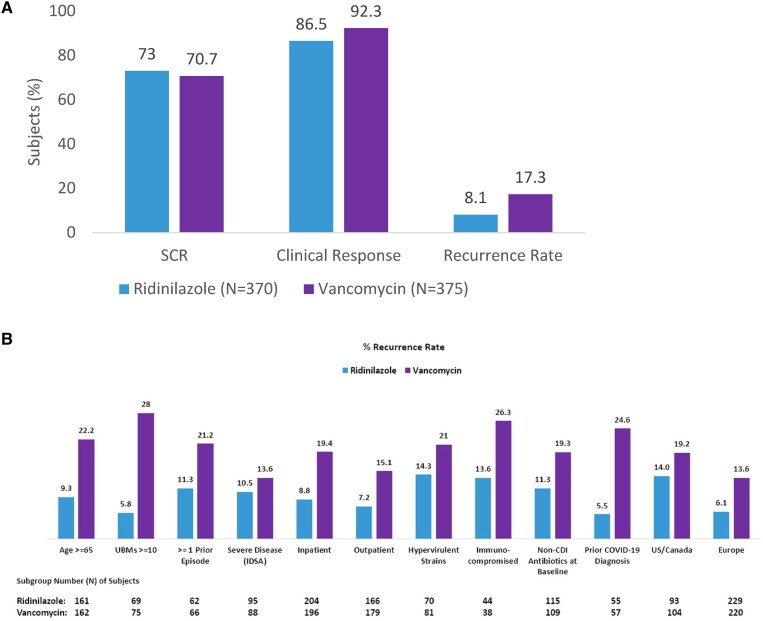
Clinical efficacy outcomes in 745 patients with CDI randomized to ridinilazole or vancomycin. *A*, Depicts SCR, clinical response, and recurrence rate in the mITT population. *B*, Depicts CDI recurrence rate in prespecified subgroups. Numbers above the boxes indicate the median percentage of SCR, clinical response, and recurrence, as appropriate. The primary endpoint was SCR, defined as clinical response and no recurrent CDI through 30 days post–end of treatment. Abbreviations: CDI, *Clostridioides difficile* infection; COVID-19, coronavirus disease 2019; IDSA, Infectious Diseases Society of America; mITT, modified intent-to-treat; SCR, sustained clinical response; UBM, unformed bowel movement.

In the mITT population, RDZ and VAN achieved an SCR rate of 73.0% (270/370) versus 70.7% (265/375), respectively (treatment difference of 2.2%; 95% CI: −4.2, 8.6; *P* = .4672) (Table 2). Accordingly, the formal hierarchical statistical test procedure was stopped, and the *P* values reported below are nominal *P* values. Clinical response rate was 86.5% in the RDZ group and 92.3% in the VAN group (treatment difference: −6.2%; 95% CI: −10.8, −1.6) (Figure 2*A*).

**Table 2.** Response Rates According to Treatment Arm: mITT Population

	RDZ (n = 370), n (%)	VAN (n = 375), n (%)	Treatment Difference, % (95% CI)
Sustained clinical response	270 (73.0)	265 (70.7)	2.2 (−4.2, 8.6)
Clinical response	320 (86.5)	346 (92.3)	−6.2 (−10.8, −1.6)
Recurrence	30 (8.1)	65 (17.3)	−9.2 (−14.1, −4.5)

Abbreviations: CI, confidence interval; mITT, modified intent-to-treat; RDZ, ridinilazole; VAN, vancomycin.

**Figure 3.**
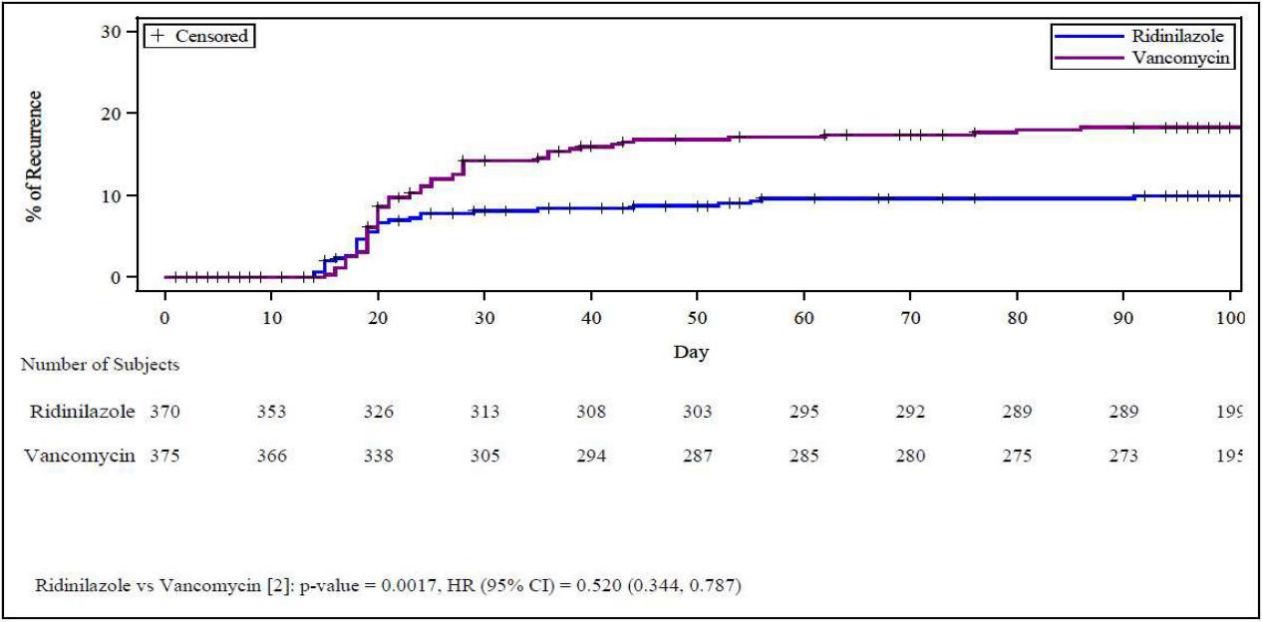
Cox proportional hazards of developing a recurrence of CDI shown as a percentage of the study population experiencing a CDI recurrence during the 100 days of study. *P* values were obtained by log-rank test stratified by randomization stratification factors, age group (<65 years or ≥65 years) and history of CDI in the past 12 months (none or 1–3 previous occurrences). HRs and 95% CIs were estimated using a Cox proportional hazards model stratified by the 2 randomization stratification factors. Abbreviations: CDI, *Clostridioides difficile* infection; CI, confidence interval; HR, hazard ratio.

Notably, RDZ resulted in a 53% reduction in recurrence rate compared with VAN. The recurrence rate was 8.1% (30/370) in the RDZ group and 17.3% (65/375) in the VAN group (treatment difference: −9.2; 95% CI: −14.1, −4.5; *P* = .0002) (Table 2, Figure 3). Subgroup analysis revealed consistent benefit for a reduction in recurrence rates for RDZ for high-risk groups, including those aged 65 years and older and those with hypervirulent strains, immunocompromised, or with COVID-19 within 30 days prior to randomization (Figure 2*B*).

### Safety

Adverse events that occurred or worsened after the first dose of study treatment through 30 days after the last dose date or that were related to study treatment (treatment-emergent adverse events [TEAEs]) occurred in 176 out of 374 patients on RDZ (47.1%) and in 178 out of 377 patients on VAN (47.2%). Overall, the majority of the TEAEs were mild or moderate in severity. The TEAEs by severity, seriousness, or leading to discontinuation of the drug or death were not different between the 2 arms (Supplementary Table 1). The incidence of serious TEAEs was 13.4% in the RDZ group and 12.5% in the VAN group. Treatment-emergent adverse events leading to discontinuation of the study drug were reported in a lower percentage of subjects in the RDZ group (0.8%) as compared with the VAN group (2.9%) in the safety population. Treatment-emergent adverse events resulting in a fatal outcome were reported in 4.0% of subjects in the RDZ group and 3.4% in the VAN group in the safety population. None of the TEAEs resulting in a fatal outcome were related to the study drug. During the entire study duration, 50 subjects died, of whom 26 (7.0%) were in the RDZ group and 24 (6.4%) were in the VAN group.

### Bile Acids, Microbiome, and Resistome

#### Changes in Secondary Bile Acids

**Figure 4.**
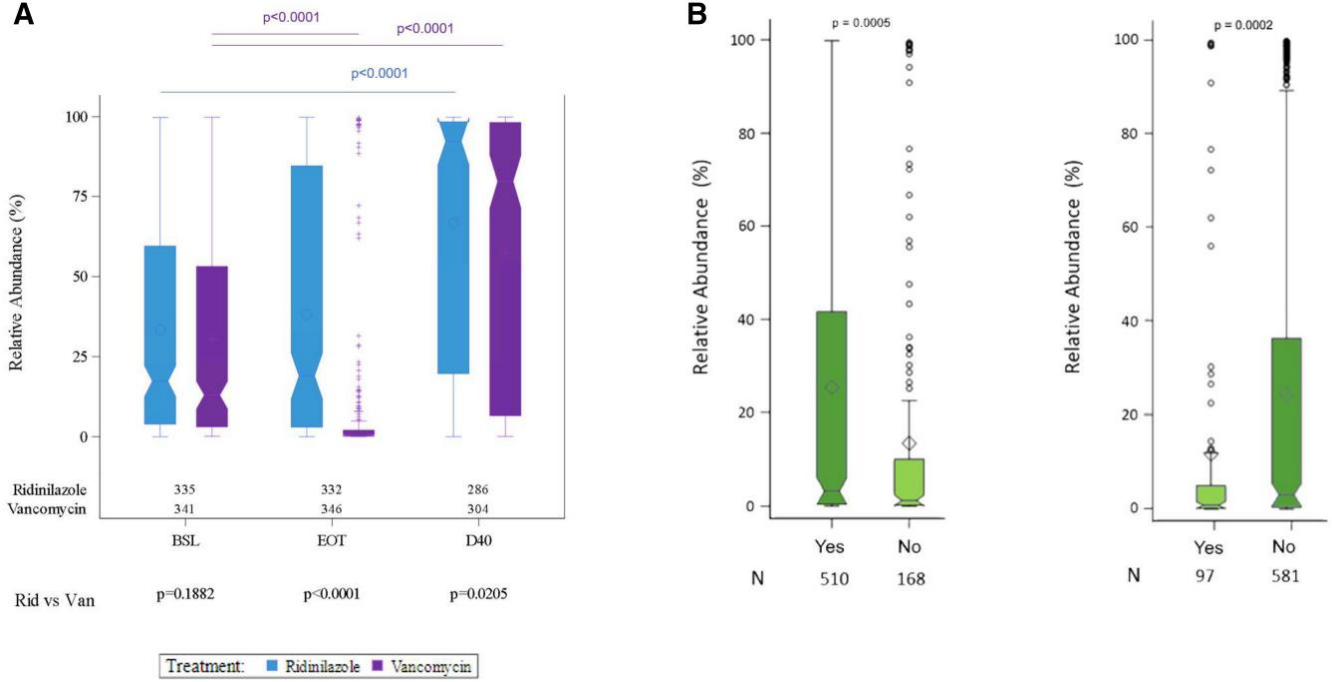
*A*, Depicts SBAs per treatment group, at BSL, EOT, and D40 post-treatment for RDZ or VAN. *B*, Depicts SBAs per treatment group including sustained clinical response (left) and recurrence (right), regardless of treatment arm randomization. Circles show the means; horizontal bars show the medians. Numbers below the boxplots indicate the number of samples at each visit for time point in RDZ and VAN treatment groups (*A*). “N” indicates number of samples included in the analysis, irrespective of treatment group. Abbreviations: BSL, baseline; D40, day 40; EOT, end of treatment; RDZ/Rid, ridinilazole; SBA, secondary bile acid; VAN/Van, vancomycin.

The relative abundance of microbiome-derived SBAs at baseline was similar in the RDZ and VAN treatment groups (median: 17.36% and 13.00%, respectively; *P* = .1882). When comparing EOT to baseline, stool SBAs increased slightly in RDZ-treated subjects (*P* = .0152) but were markedly decreased in VAN-treated subjects (*P* < .0001), resulting in higher relative abundance of SBAs in the RDZ group compared with the VAN group (18.99% vs 0.49%; *P* < .0001). Post-treatment increases in SBAs were observed in both treatment groups, but at D40, SBAs were higher in the RDZ group than in the VAN group (92.35% vs 79.69%; *P* = .0205) (Figure 4*A*).

The relative abundance of SBAs at EOT was higher in patients who achieved SCR than those who did not achieve SCR regardless of treatment arm (median values of 3.28% and 1.32%, respectively; *P* = .0005). The SBAs at EOT were higher in those who did not have recurrences compared with those who did have recurrence regardless of treatment arm (3.06% and 0.83%, respectively; *P* = .0002) (Figure 4*B*).

#### Changes in Microbiota Diversity and Composition

**Figure 5.**
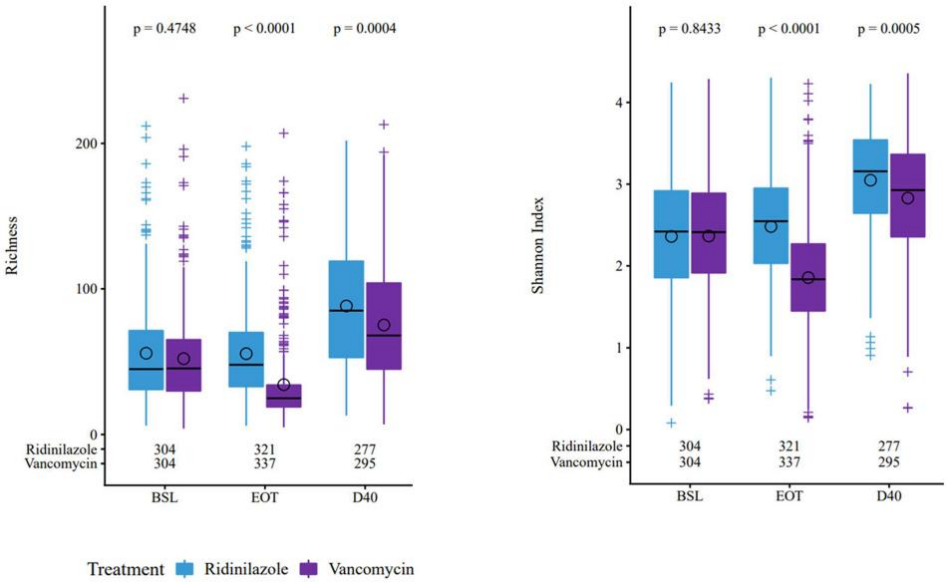
Gut microbiome differences in participants receiving RDZ or VAN as measured by richness and Shannon index (alpha-diversity). Richness refers to the total number of bacterial species present in participants receiving ridinilazole or vancomycin. The Shannon index assesses diversity by measuring the number and evenness of bacterial species between 2 groups. The higher the Shannon index, the greater the diversity in a group. Circles show the means; horizontal bars show the medians. Numbers below the boxplots indicate the number of samples at each visit for time point in RDZ and VAN treatment groups. Median richness values in RDZ and VAN groups were 45.0 vs 45.5 at BSL, 48.0 vs 25.0 at EOT, and 85.0 vs 68.0 at D40, respectively. Median Shannon index values in RDZ and VAN groups were 2.42 vs 2.41 at BSL, 2.55 vs 1.84 at EOT, and 3.16 vs 2.93 at D40, respectively. Abbreviations: BSL, baseline; D40, day 40; EOT, end of treatment; RDZ, ridinilazole; VAN, vancomycin.

As shown in Figure 5, alpha-diversity was similar at baseline for both groups. At EOT, microbiota diversity was preserved for RDZ, whereas VAN significantly worsened gut dysbiosis (median richness: 48.0 vs 25.0; *P* < .0001; median Shannon index: 2.55 vs 1.84; *P* < .0001). At D40, alpha-diversity measures were higher in the RDZ group than in the VAN group (median richness: 85.00 vs 68.00; *P* = .0004; median Shannon index: 3.16 vs 2.93; *P* = .0005).

Beta-diversity measures (Jaccard distance and Bray-Curtis dissimilarity) between paired baseline and EOT samples of individual subjects showed that RDZ treatment had a lower impact on the microbiota composition than VAN (both measures, *P* < .0001) (Supplementary Figure 2).

**Figure 6.**
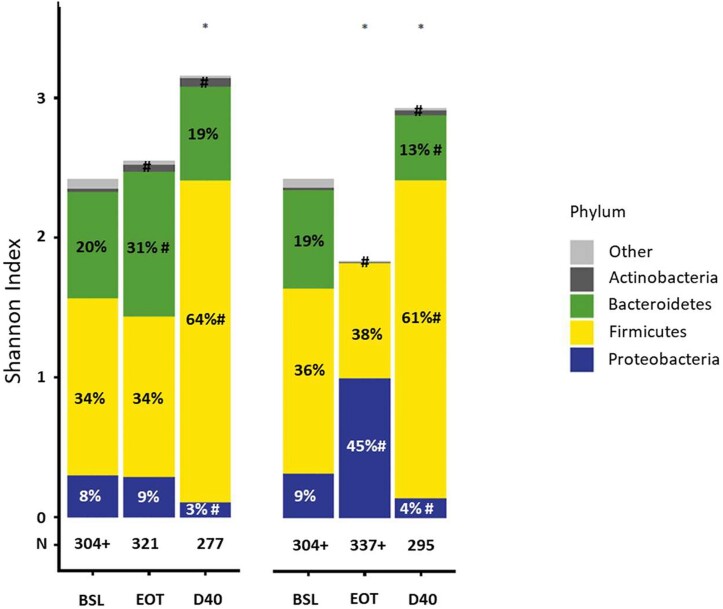
Gut microbiome differences at the phylum level in participants taking RDZ or VAN measured by metagenomic deep shotgun sequencing at BSL, EOT, or D40 post-treatment. “N” indicates numbers of samples at the indicated time points in RDZ and VAN treatment groups used for the Shannon index and phyla relative abundance analyses. Median Shannon index and median relative abundance of bacterial phyla >2% at any given time point are represented. Phyla with lower relative abundances are included in the “Other” category. Abbreviations: BSL, baseline; D40, day 40; EOT, end of treatment; FDR, false discovery rate; RDZ, ridinilazole; VAN, vancomycin. ^+^For the analysis of the relative abundance of bacterial phyla, 313 and 316 baseline samples for RDZ and VAN were used, respectively (vs 304 samples for Shannon index), and 338 EOT samples for VAN were used (vs 337 samples for Shannon index). *Significant change from baseline in Shannon index using Wilcoxon signed­ rank test comparing baseline with post-baseline visits, *P* < .05. ^#^Significant change in the phylum relative abundance compared with baseline using the FDR-adjusted *P* value from the Wilcoxon signed-rank test comparing baseline with post-baseline visits.

Significant differences in the impact of RDZ and VAN on the microbiome taxonomic composition were also noted. At EOT, RDZ resulted in the expansion of Actinobacteria (+1.23 median log_2_ fold-change [FC]; FDR-adjusted *P* < .0001) and Bacteroidetes (+0.26 median log_2_ FC; FDR-adjusted *P* = .0047), which contain many commensal species. In contrast, VAN resulted in a significant decrease in the median relative abundance of Bacteroidetes (−7.10 median log_2_ FC; FDR-adjusted *P* < .0001) and Actinobacteria (−1.11 median log_2_ FC; FDR-adjusted *P* < .0001), and a concomitant expansion in Proteobacteria (+1.72 median log_2_ FC; FDR-adjusted *P* < .0001) (Figure 6). Changes in microbiome taxonomic composition at the family level are shown in Supplementary Figure 3 and Supplementary Table 2.

#### Changes in the Resistome

**Figure 7.**
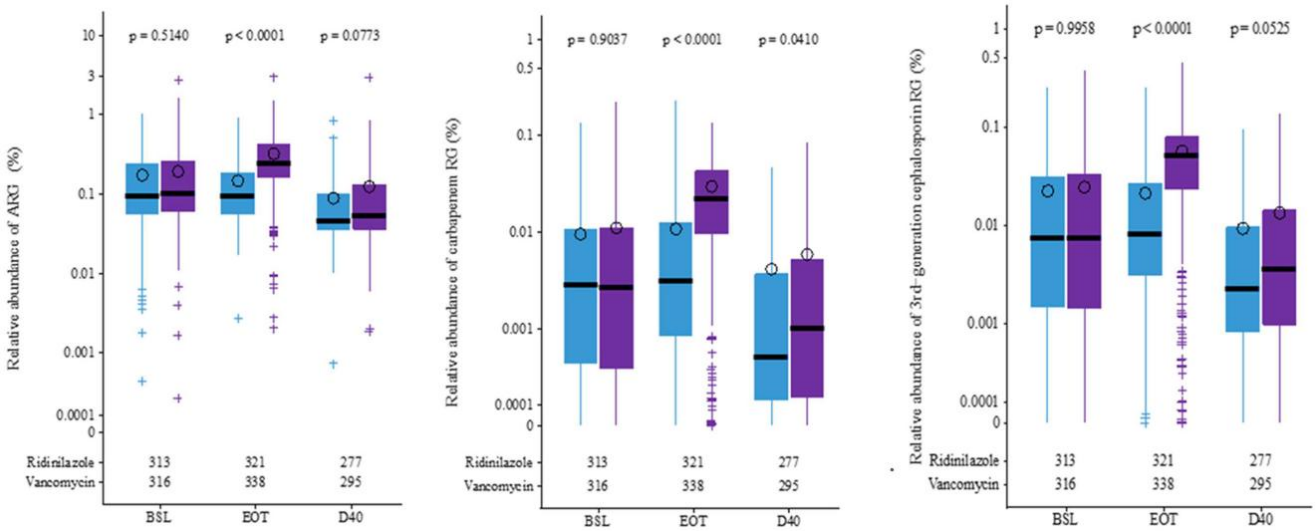
Presence of ARGs, carbapenem RGs, or third-generation cephalosporin RGs in the stool of participants taking RDZ or VAN at BSL, EOT, and D40. Circles show the means; horizontal bars show the medians. Numbers below the boxplots indicate the number of samples at each time point in RDZ and VAN treatment arms. *P* values from Wilcoxon rank-sum test to compare RDZ and VAN treatments. In RDZ and VAN groups, respectively, median relative abundances in total ARGs were 0.11% vs 0.11% at BSL, 0.10% vs 0.27% at EOT, and 0.051% vs 0.06% at D40; median relative abundances in carbapenem-RGs were 0.0031% vs 0.0029% at BSL, 0.0034% vs 0.024% at EOT, and 0.00055% vs 0.0011% at D40; median relative abundances in third-generation cephalosporin-RGs were 0.0082% vs 0.0083% at BSL, 0.0089% vs 0.056% at EOT, and 0.0025% vs 0.0039% at D40. Abbreviations: ARG, antibacterial resistance gene; BSL, baseline; D40, day 40; EOT, end of treatment; RDZ, ridinilazole; RG, resistance gene; VAN, vancomycin.

We studied the relative abundance of antibiotic RGs (ARGs) in aggregate (forming the gut resistome) and focused on RGs conferring resistance to carbapenems and to third-generation cephalosporins (3GC). No differences were noted at baseline between the 2 treatment groups. At EOT, VAN treatment led to an expansion of total ARGs, carbapenem-RGs, and 3GC-RGs. By D40, the relative abundance of total ARGs decreased to levels lower than baseline and were similar in both treatment groups; However, in the RDZ group, the relative abundance of carbapenem-RGs was lower and 3GCs-RGs trended to lower levels (Figure 7).

## DISCUSSION

Since CDI recurs in 15% to 30% of cases, successful outcomes following therapy require treating the initial episode and preventing rCDI. Central to both outcomes is the need to selectively eradicate *C. difficile* and avoid additional long-lasting dysbiosis. In this study, we show that RDZ is well tolerated, safe, and effective for the treatment of both CDI and the prevention of rCDI. reflecting the activity and selectivity of RDZ against *C. difficile*. Ridinilazole did not demonstrate superiority to VAN in SCR at 30 days post-EOT. There are several potential explanations as to why the SCR in this study was different than expected based on the phase 2 study conducted 6 years earlier that showed a higher SCR for RDZ (66.7%) over VAN (42.4%) [20]. First, the current study was carried out during the COVID-19 pandemic when the incidence of CDI decreased markedly due to major changes in healthcare delivery and infection prevention worldwide [24]. Second, a larger proportion of patients were enrolled in Europe in the global phase 3 study as compared with the phase 2 study, which enrolled a smaller number of patients and only in the United States and Canada. Third, the distribution of infecting ribotype and hypervirulent strains known to impact disease severity has shifted and decreased considerably in the past 5 years and was only 11% in our study compared to 36% in other older studies [24]. Fourth, other factors, such as the use of osmotically active compounds as the excipient for the preparation of RDZ phase 3 tablets, could have pro-diarrheagenic effects independent of its antibiotic activity and could have interfered with the assessments of response at EOT. Finally, gaps in colonic exposure to effective therapy when the administration of 1 or more of the study doses was missed (12 hours for RDZ vs 6 hours for VAN) were potentially amplified for subjects receiving RDZ.

Although RDZ did not meet the primary endpoint of superiority for SCR versus VAN, RDZ decreased the incidence of rCDI by 53% when compared with VAN, an effect that is likely due to the RDZ microbiome-sparing specificity seen in previous studies [25]. A key factor leading to rCDI is a decrease in the relative abundance of bacteria capable of resisting *C. difficile* overgrowth. A robust, diverse microbiome prevents rCDI by metabolizing BAs present in the gut (lowering concentrations of primary BAs that can promote *C. difficile* spore germination while increasing levels of microbiome-derived SBAs that can inhibit spore germination and growth), by competing with *C. difficile* for nutrients, and by producing short-chain fatty acids such as butyrate that can reduce toxin-induced colon inflammation [11, 14, 26–28]. The mechanism responsible for the 53% relative reduction in recurrences observed in the RDZ group versus the VAN group (8.1% RDZ vs 17.3% VAN) can be found in the comprehensive microbiome and metabolome studies conducted as a part of this study. This is, to our knowledge, the largest, longest (100 days post-therapy), and most extensively characterized, double-blind, prospective microbiome and metabolome study conducted in patients receiving CDI treatment. At EOT, RDZ preserved baseline microbiota alpha-diversity, had minimal impact on the baseline taxonomic composition compared with VAN, and increased the relative abundance of protective SBAs. Furthermore, RDZ did not result in an expansion of the gut resistome. In contrast, dysbiosis worsened with VAN at the expense of potentially harmful gram-negative Proteobacteria (eg, *Escherichia coli*, *Klebsiella pneumoniae*, *Klebsiella oxytoca*) and decreased the abundance of protective SBAs. Importantly, VAN was associated with an increased relative abundance of genes coding for resistance to antibiotics, notably to carbapenems and third-generation cephalosporins.

Among the currently available CDI therapies, metronidazole is no longer recommended and VAN has unacceptable rate of rCDI episodes and likely contributes to rCDI. Bezlotoxumab can help prevent rCDI but has no role in treating CDI. Fecal microbial transplant and Firmicutes spores are emerging alternatives for both CDI and rCDI but have limitations [29], and the effect can be negated when using antibiotics to treat other infections. Fidaxomicin is now considered a first-line option for the treatment of initial CDI or rCDI, in part due to its relative microbiome-sparing activity, but is still associated with a relatively high rate of recurrence [30, 31] and is not as efficacious against hypervirulent ribotype 027 [19, 32–34].

In summary, when compared with VAN, CDI treatment with RDZ did not meet the study’s prespecified superiority threshold in SCR. Treatment with RDZ preserved microbiome diversity and thus protective SBAs, resulting in a 53% relative reduction in rCDI when compared with VAN. Ridinilazole was well tolerated and had a lower rate of treatment discontinuations due to adverse events when compared with VAN. The observed reduction in rCDI is supportive of the mechanism of action of this highly selective antibiotic that has a minimal impact on the human microbiome.

## Supplementary Data

Supplementary materials are available at *Clinical Infectious Diseases* online. Consisting of data provided by the authors to benefit the reader, the posted materials are not copyedited and are the sole responsibility of the authors, so questions or comments should be addressed to the corresponding author.

## Supplementary Material

ciad792_Supplementary_Data

## Notes

***Author contributions*.** All authors had access to the data, participated in the preparation of the manuscript, saw and approved the manuscript, and contributed significantly to the work.

***Acknowledgments.*** The authors thank the patients who participated in the trials and their caregivers and Drs. Hugh C. Wang and Md. Nazmul Huda for their bioinformatics support.

***Financial support*.** This work was partly supported with US federal funds from the Administration for Strategic Preparedness and Response, Biomedical Advanced Research and Development Authority (HHS0100201700014C), and sponsored by Summit Therapeutics Inc.
