# Supplementary material for: A Randomized, Double-Blind, Phase 3 Safety and Efficacy Study of Ridinilazole Versus Vancomycin for Treatment of Clostridioides difficile Infection: Clinical Outcomes With Microbiome and Metabolome Correlates of Response
Source: Clin Infect Dis. 2024 Feb 2;78(6):1462–72. doi: 10.1093/cid/ciad792 (PMC11175683; doi:10.1093/cid/ciad792)
Supplement: ciad792_Supplementary_Data [file ciad792_supplementary_data.docx]

**Supplementary Materials**

**Figure S1. Study Schema**

**
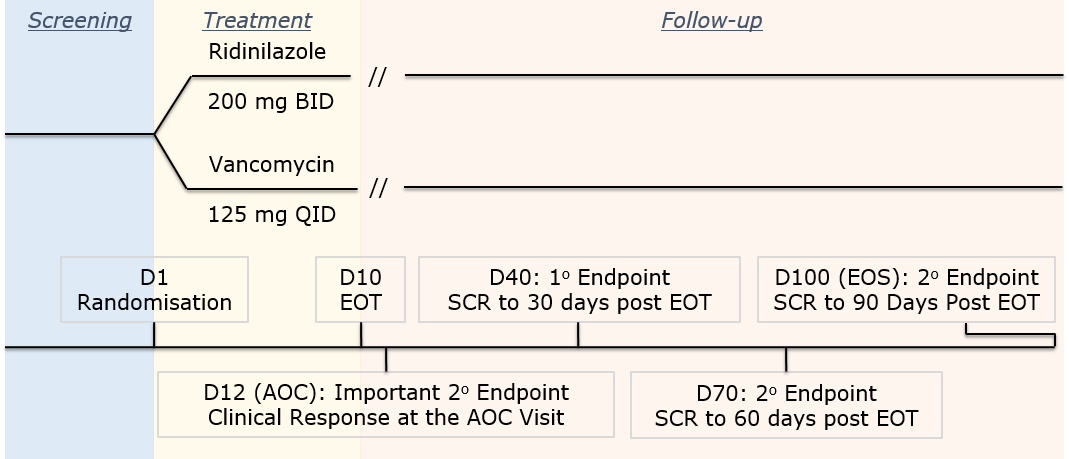
**

AOC = Assessment of Cure; BID: Twice Daily; D1: Day 1; D10: Day 10; D12: Day 12; D40: Day 40; D70: Day 70; D100: Day 100; EOT = End of Treatment; EOS = End of Study; QID: Four Times Daily; SCR = Sustained Clinical Response Assessment; 1º Endpoint: primary endpoint; 2º Endpoint: secondary endpoint.

**Figure S2. Microbial Beta Diversity Measures at End of Treatment (EOT) and at Day 40 (D40) versus Baseline (BSL) A. Jaccard Distance, B. Bray Curtis Dissimilarity**

| **A.** | **B.** |
| --- | --- |
| 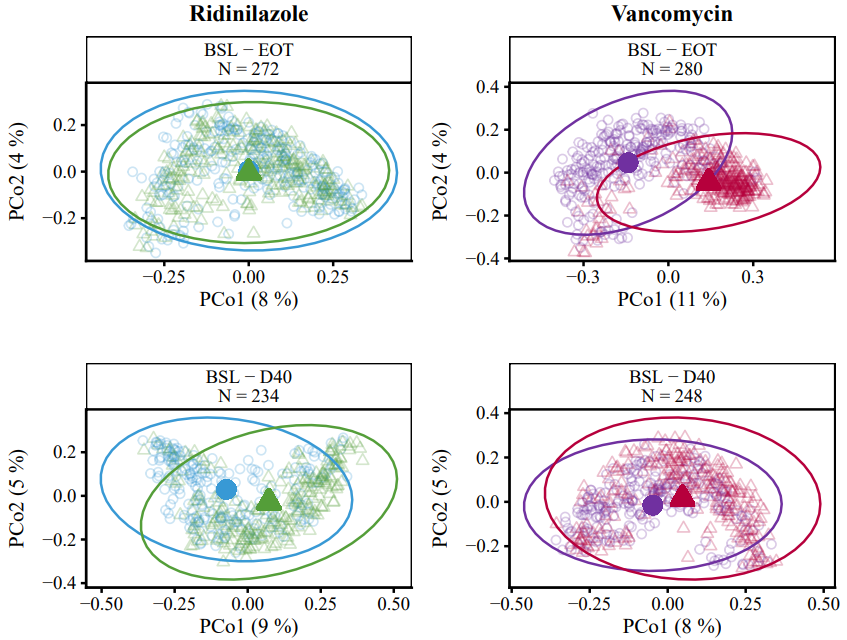 | 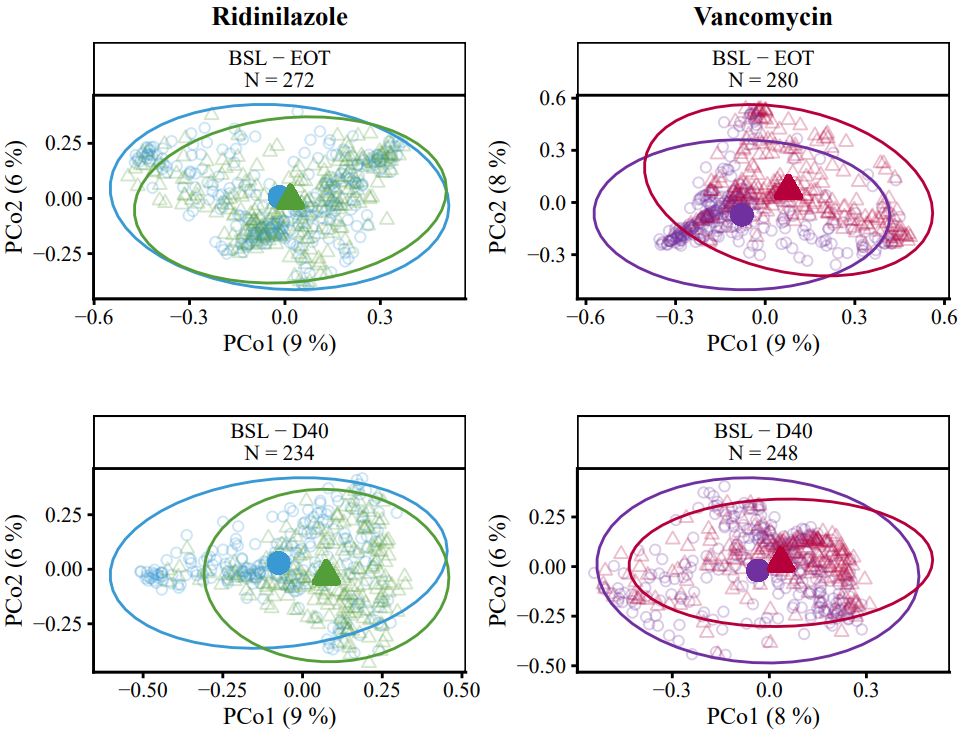 |
| \| P- values, between treatment groups \| \| \| \| --- \| --- \| --- \| \|  \| Jaccard distance \| Bray-Curtis dissimilarity \| \| BSL-EOT \| <0.0001 \| <0.0001 \| \| BSL-D40 \| 0.0017 \| 0.1179 \| | |

BSL=baseline; EOT=End of Treatment; D40=Day 40; N = number of subjects with paired samples at the indicated time-points. Principal coordinates analysis (PCoA) using Jaccard distances and Bray Curtis dissimilarity between the baseline and the post-baseline paired samples. The percentage of variation explained by the principal coordinates (PCo1 and PCo2) is indicated on the axes. The centroid, indicated with a larger solid shape, is the arithmetic mean position of all the points in each group. The ellipse represents the 95% confidence interval for each sample group. p-value from Wilcoxon rank-sum test comparing ridinilazole and vancomycin compositional differences (beta diversity) from BSL. The Jaccard distance measures the degree of microbiome dissimilarity based on the number of metagenomic species shared by two (group of) samples and the number of metagenomic species unique to each of them. The Bray-Curtis dissimilarity measures the degree of microbiome dissimilarity based on the number of metagenomic species shared by two group of samples, the number of metagenomic species unique to each of them and the metagenomic species relative abundance.

**Figure S3. Changes in the Relative Abundance of Microbial Composition at the Family Level at End of Treatment (EOT) and at Day 40 (D40) compared to Baseline (BSL).**


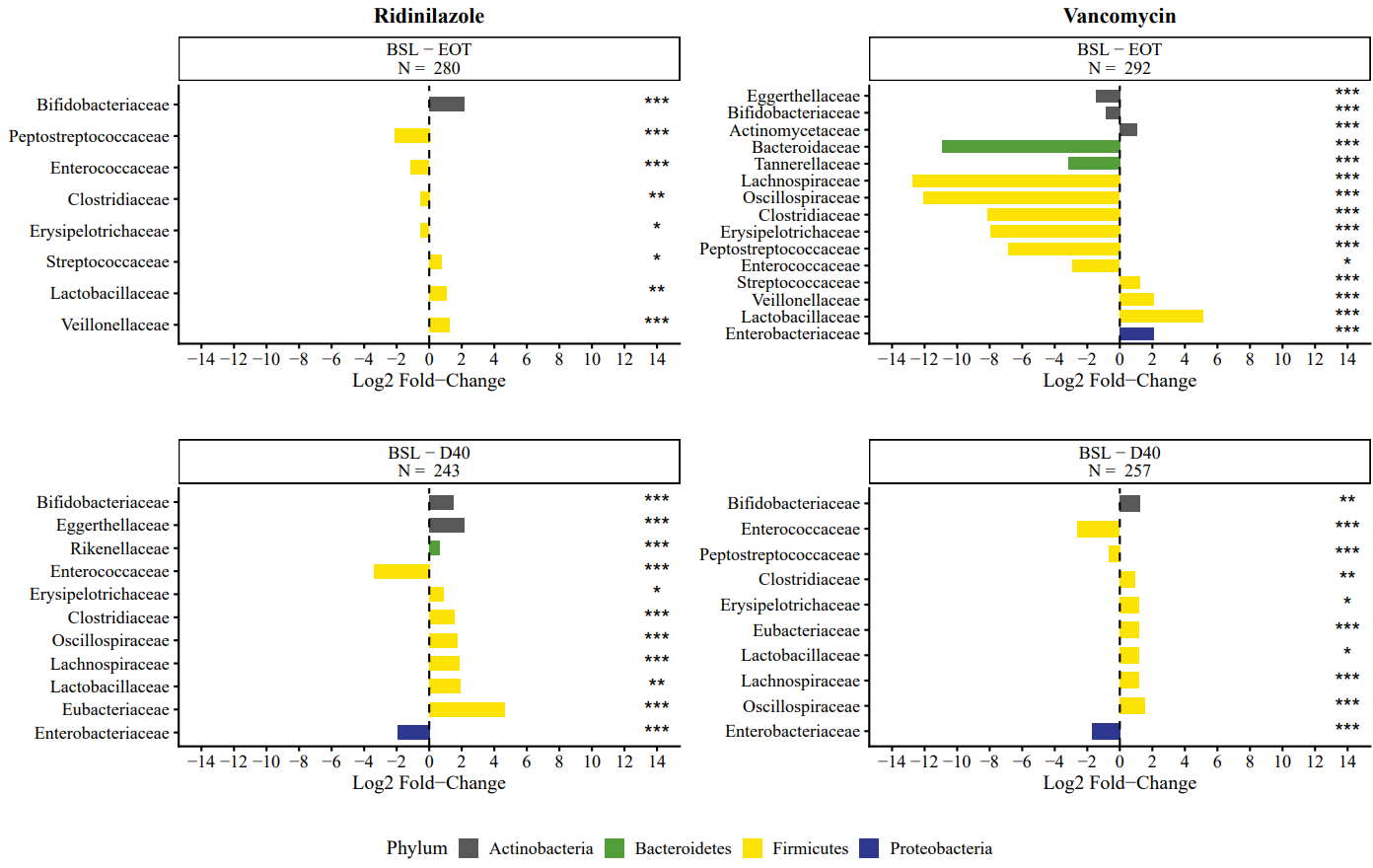


Bars represent median log2-fold changes from Baseline to End of Treatment and from Baseline to Day 40 for bacterial families whose relative abundance was statistically different between post-baseline and baseline samples and the median log2 fold change >0.5. Log2 Fold-Change from baseline = Log2 (Post-Baseline value/Baseline value). BSL=baseline; D40=Day 40; EOT=End of Treatment; N = number of subjects with paired samples at the indicated time-points. False Discovery Rate (FDR)-adjusted p values from the Wilcoxon signed-rank test comparing the relative abundance of each family at post-BSL vs BSL in ridinilazole and in vancomycin group: *: FDR p < 0.1; **: FDR p < 0.01; *** : FDR p < 0.001.

**Table S1. Overall Summary of Treatment Emergent Adverse Events (TEAE)**

|  | **RDZ (N=374) n (%)** | **VAN (N=377) n (%)** |
| --- | --- | --- |
| **Subjects with ≥ 1 TEAE** | 176 (47.1) | 178 (47.2) |
| Related TEAEs | 19 (5.1) | 18 (4.8) |
| **TEAEs by Severity** |  |  |
| Mild | 85 (22.7) | 86 (22.8) |
| Moderate | 57 (15.2) | 61 (16.2) |
| Severe | 34 (9.1) | 31 (8.2) |
| **Related TEAEs by Severity** |  |  |
| Mild | 11 (2.9) | 11 (2.9) |
| Moderate | 8 (2.1) | 5 (1.3) |
| Severe | 0 | 2 (0.5) |
| **Serious TEAEs** | 50 (13.4) | 47 (12.5) |
| Related Serious TEAEs | 1 (0.3) | 3 (0.8) |
| **TEAEs Leading to Discontinuations of Study Treatment** | 3 (0.8) | 11 (2.9) |
| Related TEAEs | 0 | 4 (1.1) |
| **TEAEs Resulting in Death** | 15 (4.0) | 13 (3.4) |

N=number of subjects in the specified population. n=number of subjects within specific category. Percentages are calculated by 100×n/N.

Adverse Events (AEs) that occur or worsen in severity after the first study treatment administration Through 30 days after the last dose of study treatment are included. Subjects experiencing more than 1 AE were counted only once at the highest severity. Adverse Events were coded using MedDRA version 24.0. Related events are those possibly/probably related or related to study treatment per investigator's judgment.

**Table S2. Changes in the Baseline Relative Abundance of Microbial Families**

|  | **Log2 Fold-Change From Baseline** | | **FDR p** |
| --- | --- | --- | --- |
| **Family** | **Ridinilazole** | **Vancomycin** |  |
| **Actinomycetaceae** |  |  |  |
| EOT | 0.00 | 1.09 | < 0.0001 |
| D40 | 0.00 | 0.00 | 0.9973 |
| **Bacteroidaceae** |  |  |  |
| EOT | 0.21 | -10.94 | < 0.0001 |
| D40 | 0.00 | 0.00 | 0.7763 |
| **Bifidobacteriaceae** |  |  |  |
| EOT | 2.13 | -0.88 | < 0.0001 |
| D40 | 1.47 | 1.24 | 0.5594 |
| **Clostridiaceae** |  |  |  |
| EOT | -0.55 | -8.14 | < 0.0001 |
| D40 | 1.54 | 0.91 | 0.2078 |
| **Eggerthellaceae** |  |  |  |
| EOT | 0.00 | -1.46 | < 0.0001 |
| D40 | 2.15 | 0.29 | 0.1358 |
| **Enterobacteriaceae** |  |  |  |
| EOT | 0.0067 | 2.10 | < 0.0001 |
| D40 | -1.95 | -1.70 | 0.4315 |
| **Enterococcaceae** |  |  |  |
| EOT | -1.15 | -2.91 | < 0.0001 |
| D40 | -3.41 | -2.60 | 0.5594 |
| **Erysipelotrichaceae** |  |  |  |
| EOT | -0.53 | -7.96 | < 0.0001 |
| D40 | 0.89 | 1.16 | 0.9434 |
| **Eubacteriaceae** |  |  |  |
| EOT | 0.00 | 0.00 | < 0.0001 |
| D40 | 4.62 | 1.16 | 0.3077 |
| **Lachnospiraceae** |  |  |  |
| EOT | 0.38 | -12.76 | < 0.0001 |
| D40 | 1.82 | 1.19 | 0.2078 |
| **Lactobacillaceae** |  |  |  |
| EOT | 1.08 | 5.12 | < 0.0001 |
| D40 | 1.90 | 1.18 | 0.7763 |
| **Oscillospiraceae** |  |  |  |
| EOT | -0.085 | -12.05 | < 0.0001 |
| D40 | 1.72 | 1.52 | 0.8954 |
| **Peptostreptococcaceae** | |  |  |
| EOT | -2.13 | -6.87 | < 0.0001 |
| D40 | -0.12 | -0.69 | 0.6570 |
| **Rikenellaceae** |  |  |  |
| EOT | 0.00 | 0.00 | < 0.0001 |
| D40 | 0.64 | 0.00 | 0.4315 |
| **Streptococcaceae** |  |  |  |
| EOT | 0.76 | 1.25 | 0.0814 |
| D40 | 0.58 | 0.29 | 0.6570 |
| **Tannerellaceae** |  |  |  |
| EOT | 0.00 | -3.19 | < 0.0001 |
| D40 | 0.00 | 0.00 | 0.2078 |
| **Veillonellaceae** |  |  |  |
| EOT | 1.25 | 2.11 | 0.0717 |
| D40 | 0.0069 | 0.20 | 0.9434 |

Median log2 fold-change between post-baseline and baseline for bacterial families represented in Figure S3, i.e., families whose relative abundance was statistically different between post-baseline and baseline samples and the median log2 fold change >0.5 in RDZ and/or VAN group at EOT and/or D40. Log2 Fold-Change from baseline = Log2 (Post-Baseline value/Baseline value). D40: Day 40; EOT: End of Treatment; FDR p: False Discovery Rate-adjusted p values indicated in this table are FDR p adjusted p values from the Wilcoxon rank-sum test comparing the change in relative abundance from baseline between ridinilazole and vancomycin treatment groups.

**Suppl. Method 1 Stool sample collection**

Subject stool samples were collected in a specimen collection container and, when applicable, brought to sites using a NanoCool® Cooling system (Cat#2-33401). At sites (or associated microbiology laboratory), stool samples were kept cold prior to and during processing which consisted of removing potential contaminants (such as blood, urine, mucus), sample homogenization and aliquoting. Sample aliquots were all prepared neat and were generally frozen within 24 hours of stool production at -70ºC or lower until analysis. Sample shipments were on dry ice.

**Suppl. Method 2 Targeted bile acid analysis of stool samples**

Concentrations of 11 bile acids (BA) in stool samples was measured by Frontage (Exton, PA, US) using a method of liquid chromatography with tandem mass spectrometry validated following the Bioanalytical Method Validation Guidance for Industry. Relative abundance of SBA was calculated as part of the total BA concentrations. Bile acids measured were 1) conjugated primary BA, glycochenodeoxycholic acid (GCDCA), taurochenodeoxycholic acid (TCDCA), glycocholic acid (GCA), and taurocholic acid (TCA), 2) primary BA, chenodeoxycholic acid (CDCA) and cholic acid (CA) and 3) SBA, alpha-hyodeoxycholic acid (α-HDCA), beta-hyodeoxycholic acid (β-HDCA), deoxycholic acid (DCA), lithocholic acid (LCA) and ursodeoxycholic acid (UDCA). Fecal samples from subjects were homogenized followed with protein precipitation and centrifugation. Chromatographic separation of the endogenous BA and their stable isotope spiked internal standards (ISs) present in the supernatants was achieved using a LC-MS/MS, Sciex API 6500+ equipped with a Shimadzu HPLC and a MACHEREY-NAGEL EC 100x2 mm NUCLEODUR C18 Gravity, 1.8µm reversed phase column. For the quantification of BA, peak areas of endogenous BA were measured against the peak areas of corresponding IS and concentrations calculated based on calibration curves generated from standards. Samples with analytes above the limit of quantification (LOQ) were diluted 10- or 50-fold and reanalyzed. For the BA statistical analyses, half the value of the lower limit of quantification was used for analytes with concentrations below the LOQ.

**Suppl. Method 3 Microbiome analysis of stool samples**

Microbiome analyses of stool samples were performed at Clinical Microbiomics (Copenhagen, DK) based on whole metagenomic deep shotgun sequencing and using their proprietary bioinformatic pipeline and microbial Metagenomic Species (MGS, an operational definition of species and sometimes subspecies) database (Nielsen et al. 2014).

Whole metagenomic deep shotgun sequencing

DNA was extracted from stool samples using the NucleoSpin® 96 Soil (Macherey-Nagel). DNA libraries were prepared using NEBNext Ultra II Library Prep Kit for Illumina (New England Biolabs) and sequenced to an average target sequence depth of ≥ 5 gigabases per sample using 2 × 150 bp paired-end sequencing on an Illumina HiSeq platform. DNA sequence reads were filtered to remove human DNA (using Bowtie2 v. 2.3.4.1) and low-quality reads and adapters (using AdapterRemoval v. 2.2.4).

Taxonomic profiling

The profiling of the high-quality non-host DNA sequenced reads was done using the Clinical Microbiomics MGS abundance profiling pipeline (acme-map, v. 4.2.1) based on Clinical Microbiomics gene catalog Hg04 that contains 14,355,839 microbial genes, MGS definitions catalog HG4.D that describes 2,095 different MGS and the MGS taxonomic annotations HG4D.2.2 (released 3 February 2022).

When calculating the fold changes and the percentage change from Baseline in the relative abundance of bacterial taxa, any “0” values (i.e., undetected) was replaced with a pseudocount corresponding to the lowest relative abundance measured divided by two.

Resistome profiling

Clinical Microbiomics gene catalog Hg04 annotation for antibiotic resistance genes (ARGs) was performed using the Comprehensive Antibiotic Resistance Database v3.1.3 and the Resistance Gene Identification (RGI) tool v5.2.0 (CARD, Alcock et al. 2020). The third generation cephalosporins considered in this analysis are used for treating human infections: cefdinir, cefditoren, cefixime, cefoperazone, cefotaxime, cefpodoxime, ceftazidime, ceftibuten, ceftriaxone, ceftizoxime.

Microbiota diversity analyses

Alpha diversity was calculated as richness (number of metagenomic species detected in a sample), and as Shannon index which in addition accounts for the abundance evenness of the species. Changes in overall microbiome community composition compared to baseline were evaluated using the beta diversity measures, Jaccard distances and Bray–Curtis dissimilarity. Jaccard distance measures the degree of microbiome dissimilarity based on the number of metagenomic species shared by two (group of) samples and the number of metagenomic species unique to each of them; additionally, Bray-Curtis dissimilarity accounts for the metagenomic species (MGS) relative abundance. Both alpha and beta diversity were calculated using the vegan R package and are based on downsized MGS data with 10,000 reads mapping to MGS per sample. Samples were downsized to equalize the detection sensitivity between them.
